# A New Anatomically Based Nomenclature for the Roots and Root Canals—Part 2: Mandibular Molars

**DOI:** 10.1155/2012/814789

**Published:** 2012-02-08

**Authors:** Denzil Valerian Albuquerque, Jojo Kottoor, Natanasabapathy Velmurugan

**Affiliations:** ^1^Private Practice, Mumbai 400050, India; ^2^Department of Conservative Dentistry and Endodontics, Mar Baselios Dental College, Kothamangalam, Ernakulam, Kerala 686691, India; ^3^Department of Conservative Dentistry and Endodontics, Meenakshi Ammal Dental College and Hospital, Alapakkam Main Road, Maduravoyal, Tamil Nadu, Chennai 600 095, India

## Abstract

Several terminologies have been employed in the dental literature to describe the roots and root canal systems of mandibular molars with no consensus being arrived at, thus far. The anatomical relation of roots and their root canals were identified and a naming system was formulated. The proposed nomenclature attempts to make certain essential modifications to the traditional approach to accommodate the naming of various aberrations presented in mandibular molars. A simple, yet extensive nomenclature system has been proposed that appropriately names the internal and external morphology of mandibular molars.

## 1. Introduction

Nomenclature refers to a set of terms used in communication by persons in the same profession that enables them to better understand one another. The comprehension of these terms aids in diagnosing and treating disease and defects of the teeth [1]. The mandibular first molar, the earliest permanent posterior tooth to erupt, is considered to be the most frequently involved tooth in endodontic procedure [2]. In its typical form, it is described as a two rooted tooth containing either three or four canals. Most commonly, mandibular molars present with two principle roots, the mesial and the distal [3, 4]. The mesial root commonly presents with two principle canals, the mesiobuccal (MB) and the mesiolingual (ML). The distal root however has two common canal configurations wherein it may contain a single canal termed as the distal (D) or may contain two separate canals, the distobuccal (DB) and the distolingual (DL). Thus, in the distal root the principle canals could either be the distal (D) or the distobuccal (DB) and distolingual (DL), as the case may be [3].

As with any tooth anatomy, mandibular molars have also been reported with numerous variations with regards to their root and root canal morphology. Variations in their root anatomy have ranged from 2 roots, as described earlier, to as many as 4 distinct roots [4–6], while canal variations have ranged from a single root canal to as many as seven root canals [7–11]. A literature search revealed that multiple atypical and diverse terms have been used in the dental literature to describe the same morphologic variation in these teeth (Table 1). Alternatively, the same names have also been used to name two nonidentical anatomical variations. For instance, a canal located between the DB and DL canal has been alternately termed as the middle distal, distal, and the third distal canal [7, 12]. Additionally, canals located in similar anatomical positions have been differentiated by using numbers as suffixes to a common name, analogous to the traditional names of their maxillary counterparts, as MB1, MB2, ML1, ML2 [7]. Also, few authors have described the variation of multiple canals within a root by merely mentioning the number of canals (e.g., 2 mesial or 3 distal canals, 2DB canals, etc.) [9, 13]. The numbers convey only the presence of an additional canal(s) with no descriptive information of the variant canal system. Of the various terminologies, the use of numbers (MB1, MB2, ML1, ML2, DB1, DB2 or 2 mesial, etc.) [14] to denote additional canals is very unusual for nomenclature. The outcome is that there is a lack of clarity in communication of the various aberrations presented in mandibular molars. This highlights the need as well as the importance of a nomenclature that takes these factors into consideration for enhanced communication, improved education, and understanding of the variations in the roots and their canal systems.

To date, no nomenclature system has been presented that simultaneously considers the relationship of the root and the root canal anatomy of mandibular molars. Thus, though there appears to be a general agreement with regards to the presence of internal and external morphological aberrations, no consensus has been arrived at for their nomenclature. The aim of this paper is to propose a new nomenclature to allow for a comprehensive anatomical description of the roots and root canals in mandibular molars.

## 2. Root and Root Canal Nomenclature

### 2.1. Nomenclature for Root Canals

**Figure 1:**
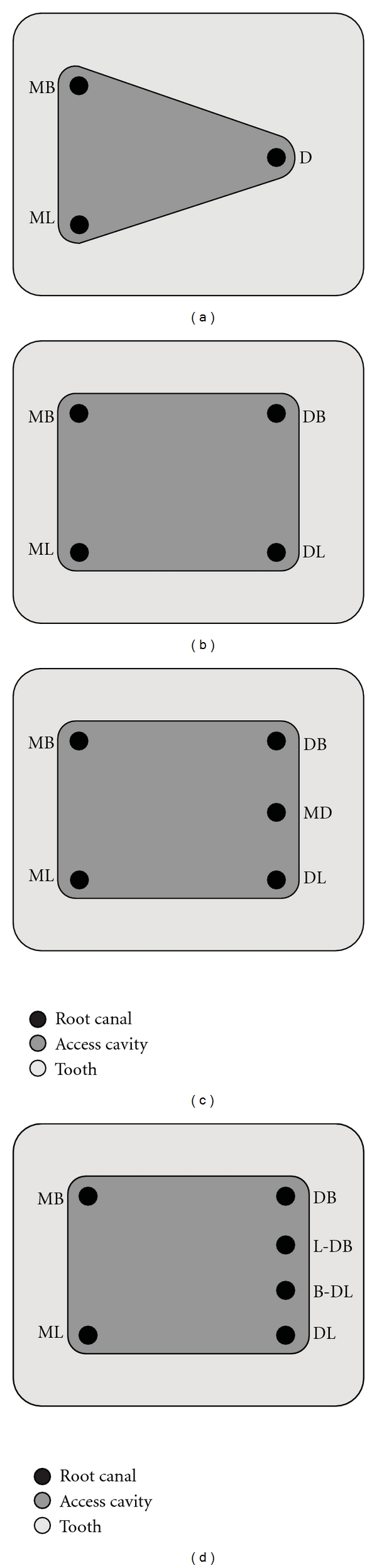
Diagrammatic representation of the pulpal floor in mandibular molars, illustrating the names of different canal configurations, according to the proposed nomenclature. The principle canals are named in accordance with the traditional approach in (a), as MB—mesiobuccal, ML—mesiolingual, and D—distal, or in (b) as MB—mesiobuccal, ML—mesiolingual, DB—distobuccal, and DL—distolingual. (c) An additional canal located in the distal root between the two principle canals is named as middle distal, MD. (d), Two additional canals located in the distal root are named as linguo-distobuccal (L-DB) and bucco-distolingual (B-DL).

1. Identification of the principle canals in two rooted mandibular molars.
  - Most commonly the mandibular molars present with two principle roots, the mesial and the distal.
  - In the principle mesial root, the principle mesiobuccal (MB) or mesiolingual (ML) canal is the canal whose orifice is located most mesially and buccally or mesially and lingually, respectively (Figure 1(a)).
  - When the principle distal root contains a single canal whose orifice is located centrally, it is identified as the principle distal (D) canal (Figure 1(a)).
  - However, when the distal root presents with its most common variation of two canals, both canals would be considered as the principle canals. They would be identified based on their respective anatomical positions and named as the distobuccal (DB) and distolingual (DL) canals (Figure 1(b)).
  - Also, the path of entrance of the canal at the level of the orifice can be used to identify the principle canals (MB, ML, D, DB, or DL), whereby the name of the canal is opposite to its path of entrance into the canal orifice.
2. An additional canal in two rooted mandibular molars.
  - When an additional canal is located between the two principle canals of the same root, the prefix “middle”, denoted as “M”, is added to describe its anatomical position between the two principle canals. The name of the additional canal would also include its mesio-distal position within the tooth, that is, “mesial” or “distal”. Thus, the canal would be named as middle mesial (MM) or middle distal (MD) canal (Figures 1(c), 5(e), and 5(f)).
3. Multiple additional canals in two rooted mandibular molars.
  - If two additional canals are contained within the same principle root(s) (i.e., 4 canals in the same root), the additional canals would be named based on their buccolingual position in relation to the nearest principle canal. The term *“bucco-”* or *“linguo-”* would be added as a prefix to the names of the principle canals that the additional canals are anatomically adjacent to. For instance, if the distal root contains four canals, all located in a similar buccolingual plane, the names of the canals would be distobuccal (DB), linguo-distobuccal (L-DB), bucco-distolingual (B-DL), and distolingual (DL) (Figure 1(d)).

### 2.2. Root Variations

**Figure 2:**
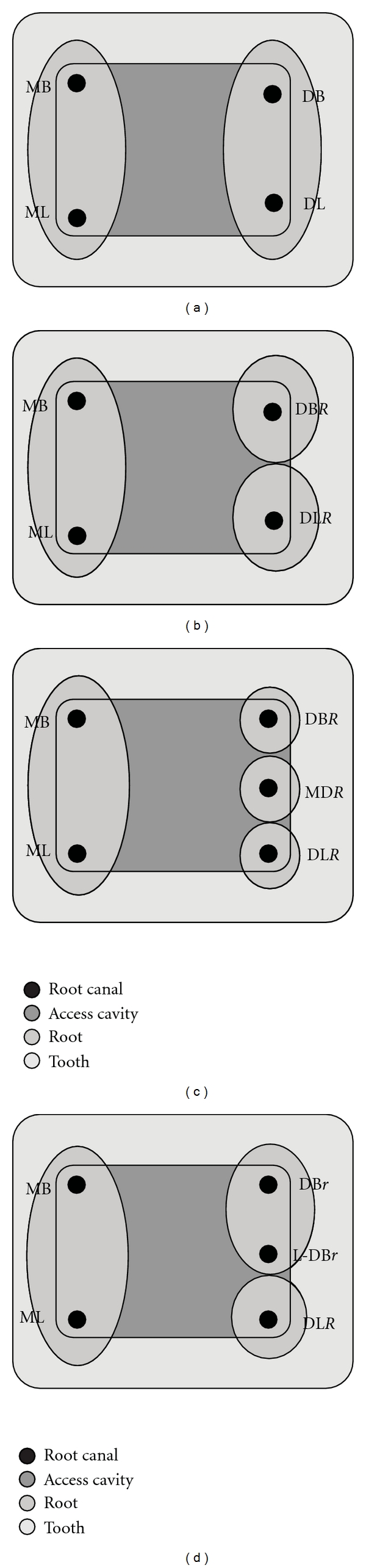
Diagrammatic representation of various root and canal configurations in mandibular molars named according to the proposed nomenclature. (a) Names of the canals would remain unaltered if the principle roots contain their principle canals; MB—mesiobuccal, ML—mesiolingual, DB—distobuccal, DL—distolingual or distal (D). (b) In mandibular molar with an additional distal root, the names of the canals in the mesial principle root will not be altered when it contains two principle canals. The canals in the two distal roots are named based on their anatomic position, as mentioned previously, and denoted by adding the suffix *“R”*. Thus, the distal root and canal variation is communicated by naming the canals as distobuccal (DB*R*) and distolingual (DL*R*). (c) When three distinct distal roots are present with each of these roots containing a single canal, the distal canals are named as distobuccal (DB*R*), middle distal (MD*R*), and distolingual (DL*R*). (d) Two distinct distal roots with the distobuccal root containing two canals, *“r”* is added as a suffix to the names of all canals contained in the additional distobuccal root, instead of *“R”*, linguo*-*distobuccal (L-DBr), distobuccal (DBr) canals.

1. Two-rooted mandibular molars
  - If all canals are located within the two principle roots, no further modification of the nomenclature is required. Thus, when the canals are named without any mention of the roots, it would signify that the canals are located in their respective principle roots. For instance, a two-rooted mandibular molar containing four principle canals would retain their names as MB, ML, DL, and DB (Figure 2(a)).
2. Three-rooted mandibular molars
  - In cases when two roots are located in place of the single principle root of that region, with each such root containing a single canal, the name(s) of the roots and their canals would be as per the anatomically based criteria mentioned above. This root variation is communicated by adding the suffix* “R”* to the abbreviated name of the canal.
  - *“R”* should be used as a suffix only to signify the root(s) other than the principle roots and when the variant root(s) contains only a single canal.
  - Thus, a three-rooted mandibular molar with two roots located distally (mesial, distobuccal, and distolingual roots) with four canals, the root, and canal configuration would be denoted as MB, ML, DB*R*, DL*R* (Figure 2(b)).
3. Four-rooted mandibular molars
  - In cases when there are three roots in place of the single principle root of that region, with each such root containing a single canal, the canals are named as per their anatomical position as mesiobuccal, middle mesial, and mesiolingual for the mesial or distobuccal, middle distal, and distolingual for the distal. Additionally, to communicate the root variation, the suffix *“R”* is added to their canal name. The root and canal nomenclature for this configuration would be denoted as MB*R, *MM*R,* ML*R* or DB*R, *MD*R, *DL*R* for the mesial and the distal, respectively (Figure 2(c)).
4. Three- or four-rooted mandibular molars with multiple canals in the additional roots.
  - In cases when any of the additional root(s) contains 2 or more canals, the name of each canal in the additional root would be based on its anatomical position within that root. The prefix bucco- (*B*), linguo- (*L*), or middle (M) would be used, as is applicable, for the naming these canals.
  - However, each of the canals in this additional root would be denoted by the suffix *“r”*, instead of the previously mentioned *“R”*. The suffix *“r”* would communicate their location within the same additional root. The root which contains these canals would be inferred from the common denominator *“r”*. Thus, two canals in the same buccolingual plane of an additional distobuccal root would be named as L-DB*r *and DB*r* (Figure 2(d)).

For ease of communication, the cited illustrations for description have been purposefully explained with relation to the distal roots and canals. However, the same criteria would also apply when morphological variations are present in the mesial roots and canals. Also, when additional canals and roots are present, they have been described to be in the same buccolingual plane as this is the pattern that mandibular molars most often demonstrate.

### 2.3. Modifications for Rare Anatomical Variations

**Figure 3:**
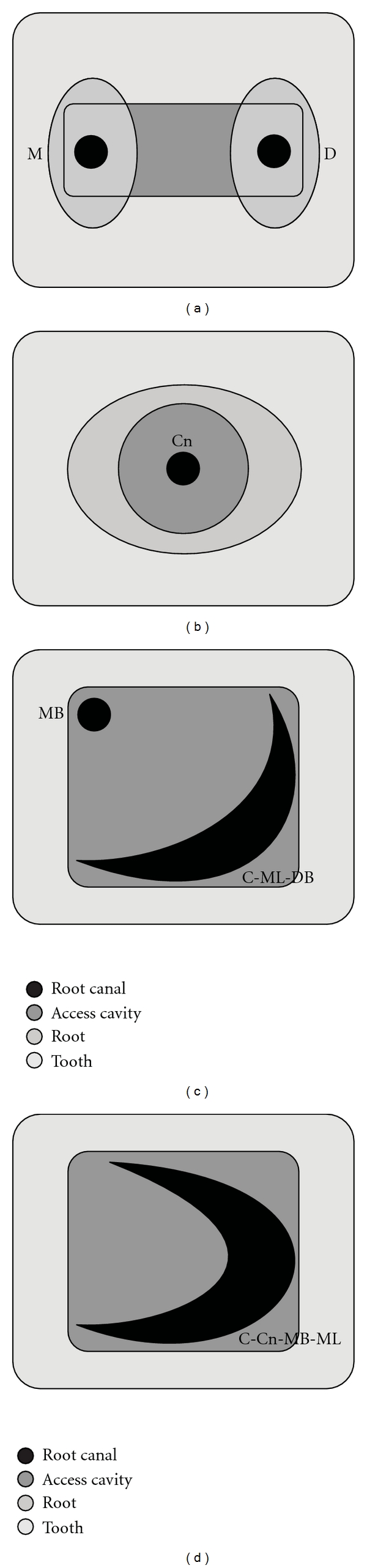
Diagrammatic representation of various root and canal configurations in mandibular molars named according to the proposed nomenclature. (a) A single canal in each principle root is named as mesial (M) and distal (D). (b) A single-rooted mandibular molar with a centrally located single canal is named as central canal, denoted as “Cn”. (c) Mandibular molar with a C-shaped canal extending from the mesiolingual (ML) to the distobuccal (DB) and a distinct mesiobuccal (MB) canal would be denoted as C-ML-DB, MB. (d), Mandibular molar with a C-shaped, centrally located canal extending from the MB to ML is denoted as C-Cn-MB-ML.

- In cases when only a single canal is located in the mesial root of a mandibular molar, the canal is named as *“mesial”*, denoted as “M” (Figure 3(a)).
- In case of a single rooted mandibular molar with a single canal, we propose that it be named as “central” canal, denoted as “Cn” (Figure 3(b)). This name appropriately describes the central location of a single canal within a solitary root.
- In cases of C-shaped canals, the prefix “C” is added to the canal name. The canal name is expanded to include the path of the C-shaped canal. For instance, a C-shaped configuration involving the ML and DB canal, with an independent MB canal, its root and canal configuration would be *C-ML-DB*, MB (Figure 3(c)). This naming pattern would also shed light on the possibility of fused roots that contain the C-shaped canal.
- When a tooth contains a single canal which is C-shaped, it is termed as C-shaped central canal and denoted as “C-Cn”. The shape, position, and extent of the canal are also included in its name, in the same order. For instance, C-Cn-MB-ML would signify a C-shaped central canal extending from the MB to the ML (Figures 3(d), 5(c), and 5(d)).

The proposed formula for naming of a root and root canal of mandibular molars, according to the present nomenclature is XR, where “X” is the anatomical location of the canal and *“R”* denotes an additional root. However, when multiple canals are located in an additional root, the formula is modified to XP*r*, where “X” is the anatomical position of the canals in relation to the principle canal (P) and *“r”* denotes the additional root that contains the multiple canals. The proposed formulas, “X*R*” and “XP*r*”, for naming of roots and root canals are applicable to various root and root canal configurations of mandibular molars, except in cases of the rare anatomical variations cited. 

## 3. Discussion

A name based on an anatomical position provides its most appropriate description. Terms that have over time gained popularity because of their simplicity are often inappropriate and imprecise. Such terms fail to anatomically describe the locations of the canals and have no parallel in scientific terminology. The present nomenclature is based on the use of anatomic terms to name the roots and their canals. This is in line with the traditional approach which employs similar anatomically based terminologies. However, in addition to the traditionally accepted terms, the proposed additional specifications help to precisely name roots and canals, inclusive of rare morphologic variations, based on anatomical considerations. This maintains the simplicity of the terms used and further widens the scope of their application and acceptance. According to Weine [23], the name of a canal is opposite to its path of entrance at the level of the canal orifice. In certain situations though, this may not be completely reliable as a criterion for naming canals. For instance, when a middle mesial canal is fusing with either the MB or the ML canal, their paths of entrance into the canal at the orifice level may vary or be more or less in the same direction. Also, multiple canals in the same root may not have a distinctively different path of entrance than their adjacent canals. This allows room for confusion when canals are named only based on their paths of entrance at the orifice level. However, as stated previously, the path of entrance of canals at the level of the orifice could be used in conjunction with their anatomical positions to locate and identify the principle canals.

**Figure 4:**
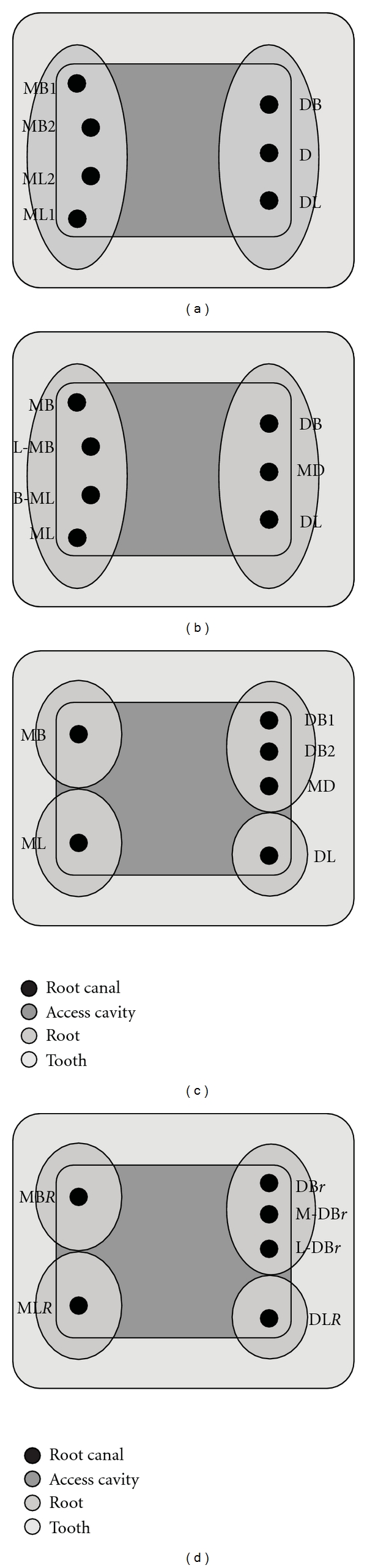
(a) Diagrammatic illustration of the access cavity of the mandibular first molar showing the locations of the seven canals contained within the principle roots and named using the traditional nomenclature. MB—mesiobuccal, ML—mesiolingual, DB—distobuccal, DL—distolingual, D—distal. (b) Naming of the canals in (a), as per the proposed anatomically based nomenclature, linguo-mesiobuccal (L-MB), bucco-mesiolingual (B-ML), MD—middle distal. (c) Diagrammatic illustration of the access cavity of the mandibular first molar showing the locations of the six canals contained within four roots and named using the traditional nomenclature. (d) Naming of canals, shown in (c), in accordance with the recommended nomenclature which clearly conveys the information of the root and canal morphology of the tooth. *“R”* is added as a suffix to the name of the canal to signify additional root(s) with a single canal (MB*R*—mesiobuccal, ML*R*—mesiolingual, DL*R*—distolingual); *“r”* is added as a suffix to the names of all the canals in the additional root containing multiple canals (L-DB*r*—linguo*-*distobuccal, M-DB*r*—middle distobuccal, and DB*r*—distobuccal canal).

Reeh [7] described the endodontic management of a two-rooted mandibular molar with seven root canals which were named as MB1, MB2, ML1, ML2, DB, D, and DL (Figure 4(a)). Although traditionally the term distal (D) has been used to describe a single canal in the principle distal root, the author termed the canal located midway between the DB and the DL canals as the distal canal. Also, the canals between the MB and the ML canals were identified using numbers as the “MB2” and “ML2” canals. This underscores the lack of clarity in the traditional approach of naming the canals of the mandibular molar based on their location. As per the proposed classification, the root and root canal morphology would be named as MB, *L-*MB, *B-*ML, ML, DB, MD, and DL (Figure 4(b)), which clearly defines the anatomical positions of these canals.

The prognosis of an endodontically treated tooth depends mainly on the adequate cleaning and shaping of the various aberrations of the root and canal system. Thus, giving adequate importance to both, the roots and their canal systems, is imperative for long-term success of endodontic treatment. The proposed system enables better communication of the root configuration of the tooth by positively including the information of the additional root(s) in the nomenclature. For instance, a mandibular first molar with four roots (MB, ML, DB, DL) containing six canals were named as MB, ML, DB1, DB2, MD, and DL [10] (Figure 4(c)). According to the proposed nomenclature, the root and canal configuration would be MB*R*, ML*R*, DL*R*, DB*r*, *M*-DB*r,* and *L*-DB*r* (Figure 4(d)). It distinctly communicates that the MB, ML, and DL canals are individual canals in their respective roots, while the DB root has three distinct canals. These instances point out to the usefulness of the proposed classification in giving a clear picture of the existing root and canal aberrancies in mandibular molars.

**Figure 5:**
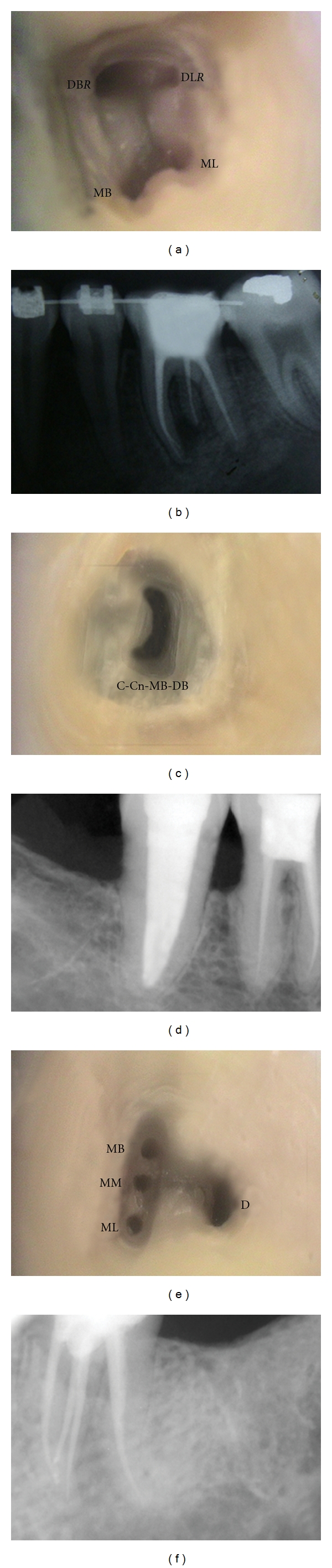
Clinical and radiographic images of anatomic aberrations demonstrating the use of the proposed nomenclature. (a, b) Pulp chamber floor showing an additional distolingual root and canal (DL*R*) and the distobuccal canal (DB*R*) in the distobuccal root; MB—mesiobuccal, ML—mesiolingual. (c) C-shaped centrally located (Cn) canal extending from the mesiobuccal (MB) to the distobuccal (DB) portion of the tooth; within a single root (d). (e, f) Three canals in the mesial root of a mandibular first molar; MM—middle mesial, D—distal.

In the proposed nomenclature, no specific mention has been made of the terms radix entomolaris (RE) [17] or radix paramolaris (RP) [18]. It is the view of the authors that each variation should be identified based on its anatomical description and without using individual names to specify each variation. Additionally, numerous other morphological variations that cannot be identified as the RE or RP have also been reported but without any specific names or a defined set of criteria for their naming. Thus, identifying these root variations as an additional DL or an MB root, along with the canal configuration it presents, is straightforward and appropriate (Figures 2(d), 4(d), 5(a), and 5(b)).

The salient features of the proposed nomenclature are its anatomical basis for location and naming of roots and canals and consideration of the root to canal relationship. It is elaborate to cover various aberrations of the root and root canal anatomy yet is simple, self explanatory, easy to understand and communicate. A certain paradigm shift has been adopted for the proposed nomenclature, but a genuine effort has been made to use the traditional naming system wherever feasible to allow for an accurate anatomical description of roots and their canals. Also, an attempt has been made to include various previously reported root and canal variations in mandibular molars within the purview of the proposed nomenclature [7, 10]. Nevertheless, given the nature of unpredictability in the endodontic field, certain aberrations could be reported in the future that may not have been covered under the scope of the present nomenclature. However, minor modifications in the form of additional criteria would enable their inclusion within the proposed nomenclature.

## 4. Conclusion

The proposed naming system is an anatomically based nomenclature which takes into account the root-to-root-canal relationship in mandibular molars.

## Figures and Tables

**Table 1:** Table summarizing the variations of roots and the canal anatomy of mandibular molars, as reported by various authors, with the numerous terms that have been used to name these aberrancies.

Root nomenclature	Root canal nomenclature	Reference
M, DB, *MD*, DL	MB, ML, DB, *MD*, DL,	[5]
M, D	MB, ML, *MD*, DB, DL	[15]
M, DB, DL	MB, ML, DB, DL, *MD or third distal *	[12]
M, D	MB, *MM*, ML, D	[16]
M, D	MB, *MM*, ML, DB, DL	[13]
M, D	M, D	[11]
*Radix entomolaris*		[17]
*Radix paramolaris*		[18]
MB, ML, D	*MB_1_, MB_2_*, *ML_1_, ML_2_*, DB, *D*, DL	[7]
MB, ML, DB, DL	ML, MB, DL, *MD*, *DB1, DB2 *	[10]
M, DB, DL	*2 mesial, 3 distal*	[19]
M, D	*4 mesial canals*, 1 distal canal	[20]
	Mesiocentral canal	[21]
M, D	MB, ML, *3rd mesial*, DB, DL	[22]
M, DB, DL	MB, ML, *2DB, 1DL *	[9]

*M*—mesial, *MB*—mesiobuccal, *ML*—mesiolingual, *MM*—middle mesial, *D*—distal, *DB*—distobuccal, *DL*—distolingual, *MD*—middle distal.
